# Return to Work after Surgical Treatment of Osteochondral Defects of the Talus: A Systematic Review and Meta-Analysis

**DOI:** 10.1177/19476035261457600

**Published:** 2026-06-04

**Authors:** Kaj S. Emanuel, Sophia van Beelen, Jari Dahmen, Julian J. Hollander, Sjoerd A.S. Stufkens, Paul P.F.M. Kuijer, Gino M.M.J. Kerkhoffs

**Affiliations:** 1 Department of Orthopedic Surgery and Sports Medicine, Amsterdam Movement Sciences, RinggoldId: 26066 Amsterdam UMC, Location AMC, University of Amsterdam, Amsterdam, The Netherlands; 2 Academic Center for Evidence based Sports medicine (ACES), Amsterdam UMC, Amsterdam, The Netherlands; 3 Amsterdam Collaboration for Health and Safety in Sports (ACHSS), International Olympic Committee (IOC) Research Center, Amsterdam UMC, Amsterdam, The Netherlands; 4 Department of Public and Occupational Health, Amsterdam Movement Sciences, Amsterdam UMC, University of Amsterdam, Amsterdam, The Netherlands

**Keywords:** allograft, articular cartilage, clinical research, clinical research ankle, clinical research cartilage and bone tissue engineering, clinical research chondrocytes or chondrogenic stem cells implantation, general

## Abstract

**Introduction:**

Return to work (RTW) is a key goal for patients treated for osteochondral lesions of the talus (OLT).

**Aim:**

The primary objective of this review is to determine the RTW after surgical treatment of an OLT.

**Methods:**

After preregistration, MEDLINE (PubMed), Embase and Cochrane Library were screened by two authors. RTW time and rate were extracted, and pooled if sufficient data was available. Two authors assessed methodological quality using the MINORS criteria.

**Results:**

A total of 18 studies were included for which 399 patients reported RTW rate and/or time. The overall methodological quality of the studies was poor. The RTW time ranged from 2 to 11 weeks post-operatively and varied across surgical techniques. The overall RTW rate was 93%, with arthroscopic procedures averaging 96%, and open procedures averaging 91% RTW.

**Conclusion:**

After surgical treatment of OLTs, 93% of patients returned to work. Time to RTW ranged from 2 to 11 weeks, although reporting was heterogeneous and methodological quality was generally low. Future studies should include RTW as a core outcome and report work-related restrictions more consistently.

## Introduction

Osteochondral lesions of the talus (OLTs) are defects of the articular cartilage and its subchondral bone.^1-3^ These lesions primarily present after an ankle trauma, such as a sprain or fracture.^3-5^ Patients often experience deep ankle pain during activity, however also a decreased range of motion (ROM), stiffness, catching, locking and swelling may be present.^6-8^ These symptoms may limit the ability to perform activities of daily living (ADL). As OLT patients are generally from a young and active population, the ability to work may also be affected in a relatively large proportion of patients. This is of great importance, as work is considered one of the key determinants of quality of life and is strongly related to both mental and physical health.^9^ Inability to work may adversely affect career progression and financial security. Furthermore, the diminished contact with colleagues decreases the social health and the sudden spare time might cause boredom.^10^ Moreover, patients not returning to work also pose a large financial burden for society, especially in young patients.^11^

Studies on OLTs often assess treatment outcomes using clinical parameters, for example using Patient Reported Outcome Measures (PROMs). These PROMs often focus on constructs such as function, pain, and quality of life (QoL).^12^ Additionally, return to sport (RTS) is often considered as secondary outcome variable.^13^ However, return to work (RTW) is not often reported upon, while it is of major influence in daily practice for patient and clinicians when opting for a specific treatment option.

Therefore, it is the primary aim of the present systematic review to determine the RTW after surgical treatment for an OLT.

## Methods

This systematic review protocol was registered prospectively in the PROSPERO database (registration number: CRD42023469471). The study was conducted in accordance with the Preferred Reporting Items for Systematic Reviews and Meta-analyses (PRISMA) guidelines.^14^

### Search Strategy

The databases MEDLINE (PubMed), EMBASE (Ovid), and Cochrane Library (Wiley) were searched up to May 1^st^ 2025 using two main keywords “Osteochondral defects” and “Talus” and all synonyms. The full search strategy can be seen in the Appendix. Synonyms of work were not included in the search as they are not always primary outcomes, and may therefore not appear in the title or abstract, even if measured as secondary outcome variables. Title and abstract screening was performed followed by full-text screening by two observers. If patient overlap was suspected in different studies, the author was contacted for further investigation.

### Eligibility Criteria

All cohort studies with at least five adult (≥18 years) patients, surgically treated for osteochondral lesions of the talus and explicitly reported RTW time and/or rate were eligible for inclusion. Students were considered as workers, and return to study therefore as RTW. For studies that included patients with multiple diagnoses or multiple interventions, it was necessary to be able to analyse the results of each diagnosis or intervention separately to include the study. Case reports were excluded as RTW rate cannot be calculated from data that is not systematically recorded and reported. Articles written in other languages than English, Dutch, German, French or Spanish were excluded.

### Quality Assessment

Methodological quality was assessed for all studies using the MINORS (Methodological Index for Non-Randomized Studies) criteria. Each study was graded on methodological quality by two independent reviewers after which any conflicting outcomes were resolved by a discussion.

### Data Extraction

Extracted variables were title, author and year. Patient characteristics comprised the number of patients, gender, age (mean, standard deviation (SD), range), lesion surface area (mean, SD, range), lesion size (length, width, depth; mean, SD, range), type of surgical intervention, follow-up duration (mean, SD, range), type of work, return to work time, return to work rate, and any restrictions or adaptations after return to work.

Surgical treatment procedures were classified based on the categorization by Hollander, et al^15^

### Outcome Measures

The primary outcome measure of the present study was the RTW, expressed in either rate or time, as recommended by the core outcome set.^16^ RTW rate is defined as the proportion of patients that resumed work at any level within the follow-up of the study following surgical intervention, expressed as a percentage of the total study population. The RTW time is defined as the duration from the surgical intervention to the patient’s RTW at any level, expressed in weeks or months.

### Statistical Analysis

Descriptive statistics were used to present quantitative data as patient characteristics. If possible, means were pooled weighted for sample size. RTW time may be given in mean ± standard deviation or median with a range. For homogeneity in showing the results, the median values were transformed to mean values following Luo, et al.^17^

All analyses were performed in Excel (Microsoft Corp., Redmond, WA, USA; version 2508).

## Results

**Figure 1.**
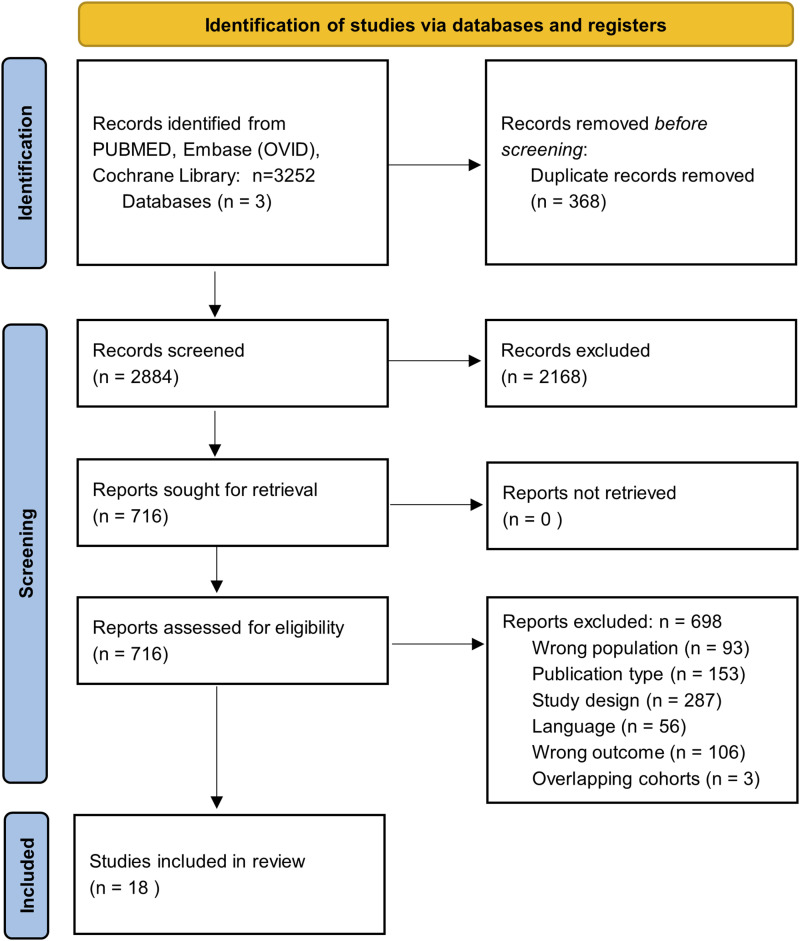
PRISMA flowchart of study selection

The search strategy resulted in 2884 unique articles of which 18 were eligible for inclusion. A PRISMA flowchart of the study selection inclusion process is available in Figure 1.

Patient overlap was established in three of the studies.^18-20^ Correspondence with the author confirmed that patients of these studies were all analyzed in another article which is included, the other three were therefore excluded.^21^ Raw data from Reilingh, et al^22^ was available allowing calculation of the mean RTW time. Following correspondence, no significant differences were observed between the intervention groups, therefore they were combined in analysis. Ogilvie-Harris conducted two studies^23,24^ with distinct patient groups based on inclusion criteria and demographics, therefore were both included.

### Study Characteristics

A total of 399 patients were included. The weighted mean of the 17/18 studies that reported age was 36.1 years (range 18 – 70 years). The minimum mean follow-up (FU) time of all articles was 12 months, the weighted mean FU time of 17 articles that reported the mean was 44.8 months (Table 1).

**Table 1.** Included Articles With the Performed Intervention, Number of Patients (#pt) and the Follow-up Time Given in Months (unless Stated Otherwise). SD = Standard Deviation. *Minimum Follow-up of 12 months was Used, However no Mean was Reported in This Study

Author	Year	Intervention	Arthroscopic	#pt	Follow-up time (months)		
			Yes/No/Mix		Mean	SD	Range
Di Cave	2017	Matrix-assisted bone marrow stimulation (TruFit Plug)	No	12	90	​	78-104.4
Ebskov	2019	Metal implants (HemiCAP®)	No	32	50	18.9	11.5-81.4
Ettinger	2017	Metal implants (HemiCAP®)	No	10	43.5	35.51	9.0-69
Jackson	2018	Osteo(chondral) allograft transplantation therapies	No	30	21	​	10-44
Kim	2020	Osteo(chondral) autograft transplantation therapies	No	32	19.5	13.3	min. 6
Koulalis	2002	Cartilage Implementation	No	8	17.6	​	8-26
Kreuz	2006	Osteo(chondral) autograft transplantation therapies	No	13	42	​	​
Lambers	2021	Bone marrow stimulation therapies	Yes	60	77	13.2	​
Loomer	1993	Multiple	Mix	38	25	​	12-120
Lopez	2019	Cartilage Implementation	Mix	24	24	​	​
Ogilvie-Harris1	1999	Bone marrow stimulation therapies	Yes	8	38	​	24-63
Ogilvie-Harris2	1999	Bone marrow stimulation therapies	Yes	33	89	​	24-219.6
Pearce	2012	Matrix-assisted bone marrow stimulation (TruFit Plug)	No	6	>12*	​	​
Reilingh	2016	Bone marrow stimulation therapies	Yes	68	12	19.17	​
Schuman	2002	Bone marrow stimulation therapies	Yes	35	58	24-132	​
Tosun	2021	Multiple	No	6	32.6	​	20-53
Vuurberg	2018	Metal implants (HemiCAP®)	No	31	61	18	​
Zhu	2016	Multiple	No	13	25.4	​	18-48

### Methodological Quality

Only one^22^ out of 18 studies had a comparative research design, and scored 16 out of 24 on the MINORS. Six studies had retrospective data collection, five were prospective and for the remaining seven, this was unclear. The non-comparative studies had an average MINORS score of 7.4 (range 5-12) out of a total of 16 points. A full overview of the scores is available in the Appendix.

### Return to Work Reported Outcomes

Only two of the included studies^21,25^ reported both the rate and time of RTW, both studying the metal implant intervention, with the remaining only reported either RTW rate or time.

### Return to Work Rate

**Figure 2.**
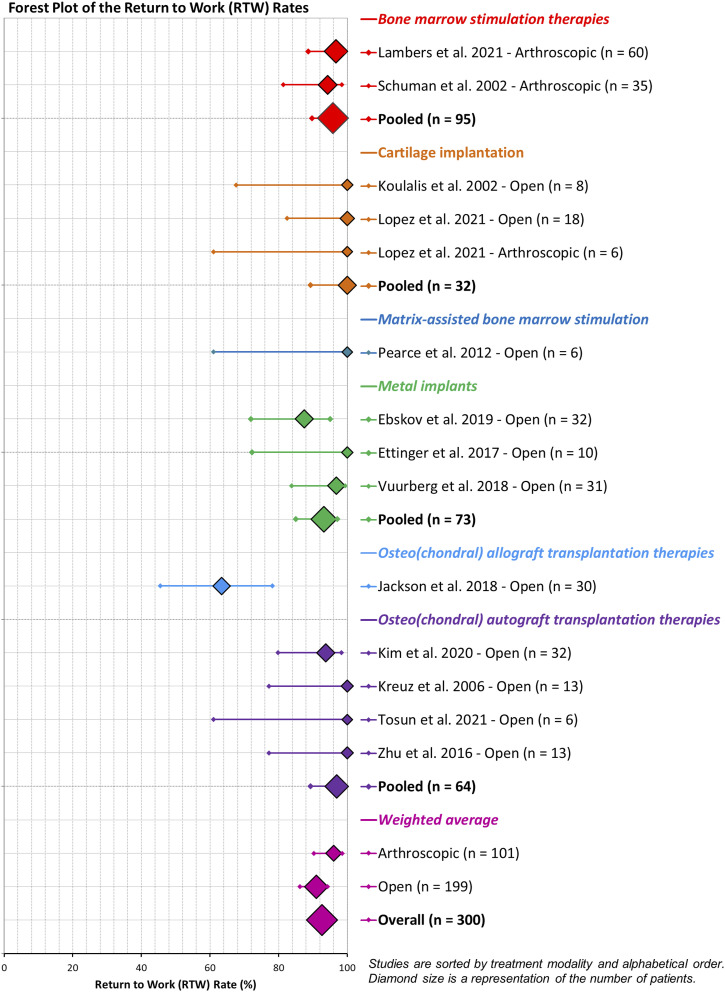
Forest plot of the RTW rate, categorized by treatment type

RTW rates were reported by 14/18 studies^21,25-36^ (n=300, Figure 2), of which three studies also reported the pre-surgery work rate. The RTW ranged from 63% to 100%, resulting in overall weighted RTW rate of 93% (278/300). The lowest RTW rate of 63% was reported by Jackson, et al.,^27^ who reported on 30 active military after osteochondral allograft transplantation. Six studies reported a RTW rate of 100%.^25,29,30,33,35,36^ When pooled by treatment modality, RTW rates were 96% (91/95) for bone marrow stimulation, 100% (32/32) for cartilage implantation, 100% (6/6) for matrix-assisted bone marrow stimulation, 93% (68/73) for metal implants, 63% (19/30) for osteo(chondral) allograft transplantation, and 97% (62/64) for osteo(chondral) autograft transplantation (Figure 2). The arthroscopic techniques (n=101) had a RTW rate of 96%, and the open techniques (n=199) showed a RTW rate of 91%. Only three studies^21,23,26^ reported the pre-operative work status, which ranged from 72-82%. However, none of those studies mentioned if the pre-operative work status was affected by the OLT.

### Return to Work Time

**Figure 3.**
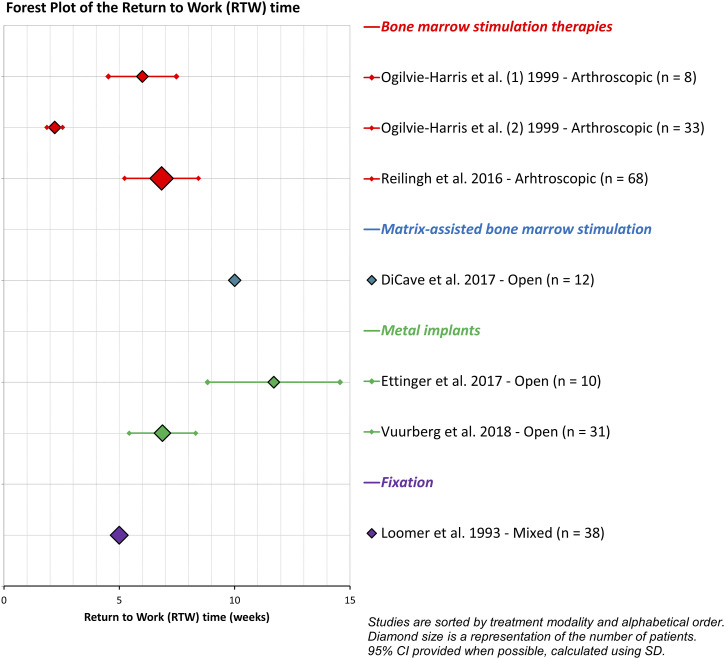
Forest plot of the RTW time, categorized by treatment type

Seven studies^3,21-25,37^ reported the RTW time (n=200, Figure 3). For patients receiving metal implantation, mean RTW times of 6.9 and 11.7 weeks were reported.^6^ For Bone Marrow Stimulation (BMS) therapies, this ranged from 2.2 to 8.1 weeks. The shortest RTW time of 2.2 weeks was reported by Ogilvie-Harris^24^ after BMS in 33 patients. Considering the between-study heterogeneity and lack of complete data (only five studies reported standard deviations), no pooling of data was conducted.

### Secondary Outcomes

Of the 14 studies that reported RTW rates, eight studies provided insight into remaining restrictions at work after return. Koulalis, et al.^29^ (N=8) and Ettinger, et al.^25^ (N=10) both reported that all patients returned to their previous work after their cartilage implantation and metal implant treatment, respectively. Within the BMS therapies group,^31^ reported that only one out of 60 patients worked less due to persistent pain, seven patients worked less due to other reasons while four patients worked more because of reduced problems. The study of Schuman et al^34^ (n=34) found that five patients experienced limitations or inability to work. Reilingh, et al^22^ included in their definition of RTW that similar work activities after surgery are reached, resulting in work at similar levels after the RTW time of 6.8 weeks.

Only Jackson, et al.^27^ reported that of the military population that received allograft transplantation, 33% returned to full military duty without running limitations, and 63% were able to remain active within the military. After autologous transplantation, Kreuz et al.^30^ found that all patients returned to their previous work.

## Discussion

This systematic review aimed to evaluate RTW rates and times following surgical treatment of OLTs. The main findings were that the average reported RTW rate after surgical treatment was 93%, with 96% for arthroscopic treatment, and 91% for open procedures. Mean RTW times ranged from 2-11 weeks, with insufficient data availability to compare between surgical techniques. Secondarily, we found that the majority of the patients returned to previous level of work, but functional limitations after returning to work were scarcely reported.

The mean RTW times in this review ranged from 2-11 weeks, with the longest RTW times reported on metal implants and matrix scaffolds. In a systematic review in 2020, the average RTW time after knee cartilage repair was 4.8 months,^38^ which is significantly longer. This is possibly because the patients in the included studies in that review were mainly treated using autologous chondrocyte implantation (ACI), which demands a long period of weight reduction (often up to 12 weeks^39^), after a two-step procedure with six weeks in between. ACI is frequently applied for OLT treatment as well,^40^ but no literature on the RTW rate or time could be identified after ACI of the talus. The results of this review are therefore possibly an underestimation for this population.

When pooled by treatment modality, RTW rates were high (≥93%) across bone marrow stimulation, cartilage implantation, matrix-assisted bone marrow stimulation, metal implants, and osteo(chondral) autograft transplantation. The exception was osteo(chondral) allograft transplantation, with a pooled rate of 63% (19/30), which was based on a single study in an active military population. This wide range likely reflects differences in patient selection — in particular lesion severity, indication for the procedure, and the physical demands of the cohort — rather than a direct effect of the procedure itself. Pooled estimates for the smaller subgroups (in particular allograft and matrix-assisted bone marrow stimulation, each based on a single study) should therefore be interpreted with caution.

The heterogeneity in reported RTW rates and time may partly be explained by the differences in treatment modalities. However, insufficient data could be gathered to test the between-group differences in RTW time. At the extremes, the longest RTW times were reported by Ettinger, et al.,^25^ who used metal implants, which is indicated for relatively severe and large (secondary) defects.^21^ Vuurberg et al.^21^however reported only 7 weeks of RTW time after implantation of the same metal implants (HemiCap®). Di Cave, et al.^3^ reported 8 weeks RTW time in sedentary patients, and 12 weeks for physically demanding jobs, although the distribution of these groups was not specified.

The shortest RTW time of 2 weeks was found by Ogilvie-Harris and Sarrosa,^24^ who applied arthroscopic nettoyage in patients with primary defects. In a separate case series using the same treatment in patients with failed previous surgery, the mean time to RTW was 6 weeks. This indicates that previous surgery may be a delaying factor in RTW.

Regarding RTW rates, most studies reported rates above 90%, with exception of the studies by Jackson and Ebskov. Jackson, et al^27^ found in a military population that only 63% of the 30 patients treated with allograft transplantation remained in active military duty. This indicates that for physically very demanding professions, RTW rates may be lower than the general population. Ebskov et al^26^ found a 88% RTW rate at 50 months after treatment with a metal implant, which can be due to a relatively severely affected population, as metal implants are seen as a last-resort treatment option. Notably, the pre-operative work rate in their population was 79%.

The average RTW rate for arthroscopic procedures was 96% and 91% for open procedures. Open procedures for osteochondral lesions of the talus are generally associated with lower RTW rates, as they are more invasive, often require malleolar osteotomy, and involve longer periods of immobilization and rehabilitation. Consequently, these procedures are associated with increased pain, slower recovery, and delayed weight-bearing. Additionally, open surgery is typically reserved for more severe or complex lesions, which inherently have worse functional outcomes compared with those treated arthroscopically.^8^

## Limitations

Although some variables were suggested as influencing the RTW time (e.g. previous surgery, surgical technique, physically demanding jobs), it was not feasible to quantify the effect size of each variable. In most studies, the RTW was a secondary outcome variable at best, with results frequently limited to general remarks like “all patients returned to work at final follow-up”. This could lead to a selection or reporting bias, as also measured by the MINORS score.

Not only the type of surgery, but also the pre- and postoperative (vocational) rehabilitation might have a strong effect on RTW rates and time. In the description of the included studies, however, little information was found about the perioperative care that might enhance RTW. Studies in orthopaedic surgery showing the added value of this so-called work-integrated care on RTW are available for lumbar disc herniation,^41^ hip and knee arthroplasty^42^ and lumbar fusion surgery.^43^

### Clinical Relevance

The RTW rate and duration provides patients with insight into their chances of returning to work, offering them a sense of perspective and aligns with personal goals of patients. This is particularly important, as unemployment can also impact patients’ mental well-being such as increased chance of depression and anxiety.^44^ Physicians can utilize these metrics to facilitate shared decision-making with patients, including discussions about the timing of interventions.

### Future Perspectives

The RTW rate and time are underreported and their significance for patients is underestimated. While return to sport has become a standard outcome measure in literature,^13^ the importance of work for quality of life is likely as impactful as sport participation.^45^ Future studies should preferably report the number of patients employed pre-operatively, specifying full-time or part-time and the type of work (physical or sedentary).^16^ Also any reductions in work hours prior to surgery related to the OLT should be noted. Post-operative data could include the RTW time and rate, the follow-up duration, and whether patients resumed their previous work, including hours and any changes in job function.

## Conclusion

In the available literature, 93% of patients with an osteochondral lesion of the talus returned to work after surgical treatment, with 96% after arthroscopic procedures and 91% after open procedures. The time to RTW ranged from 2-11 weeks, with insufficient data available to test any differences in RTW time based on surgical procedure.

## Appendix

### Appendix A

#### Search Strategy

**Table:**

#	Pubmed search
1	“Osteochondritis Dissecans”[Mesh] OR osteochondritis dissecans[tiab] OR osteochondrosis dissecans[tiab] OR osteochondrolysis[tiab]
2	(osteochondral[tiab] OR chondral[tiab] OR transchondral[tiab] OR cartilage*[tiab]) AND (defect*[tiab] OR lesion*[tiab])
3	OCD[tiab] OR OLT[tiab]
4	#1 OR #2 OR #3
5	“Talus”[Mesh] OR talus[tiab] OR talar*[tiab] OR ankle[tiab]
6	#4 AND #5
#	Embase search
1	osteochondritis dissecans/or (osteochondritis dissecans or osteochondrosis dissecans or osteochondrolysis or OCD or OLT).ti,ab,kw. or ((osteochondral or chondral or transchondral or cartilage*) and (defect* or lesion*)).ti,ab,kw.
2	talus/or (talus or talar* or ankle*).ti,ab,kw.
3	1 and 2
4	limit 3 to conference abstract status
5	3 not 4
6	animal/not human/
7	5 not 6
ID	Cochrane Search	
#1	(osteochondritis dissecans or osteochondrosis dissecans or osteochondrolysis or OCD or OLT):ti,ab,kw	
#2	((osteochondral or chondral or transchondral or cartilage*) and (defect* or lesion*)):ti,ab,kw	
#3	#1 or #2	
#4	(talus or talar* or ankle*):ti,ab,kw	

### Appendix B

#### Critical Appraisal

**Table:**

Author	A clearly stated aim	Inclusion of consecutive patients	Prospective collection of data	Endpoints appropriate to the aim of the study	Unbiased assessment of the study endpoint	Follow-up period appropriate to the aim of the study	Loss to follow up less than 5%	Prospective calculation of the study size	An adequate control group	Contemporary groups	Baseline equivalence of groups	Adequate statistical analysis	Total
Di Cave, 2017	2	0	0	2	1	2	​	​	​	​	​	​	**7**
Ebskov, 2019	2	2	0	2	0	2	0	0	​	​	​	​	**8**
Ettinger, 2017	2	2	0	2	0	1	0	0	​	​	​	​	**7**
Jackson, 2018	2	0	0	2	1	2	0	0	​	​	​	​	**7**
Kim, 2020	1	1	0	2	0	2	0	0	​	​	​	​	**6**
Koulalis, 2002	0	1	2	1	0	2	0	0	​	​	​	​	**6**
Kreuz, 2006	1	1	0	1	0	2	0	0	​	​	​	​	**5**
Lambers, 2021	2	2	2	2	1	2	1	0	​	​	​	​	**12**
Loomer, 1993	1	1	0	0	0	2	1	0	​	​	​	​	**5**
Lopez, 2019	2	2	2	2	0	2	​	0	​	​	​	​	**10**
Ogilvie-Harris1, 1999	1	1	0	1	0	2	0	0	​	​	​	​	**5**
Ogilvie-Harris2, 1999	1	1	0	1	1	2	0	0	​	​	​	​	**6**
Pearce, 2012	1	1	0	2	1	2	0	0	​	​	​	​	**7**
Reilingh, 2016	2	1	2	2	2	2	1	0	2	2	2	2	**20**
Schuman, 2002	2	1	2	2	0	2	1	0	​	​	​	​	**10**
Tosun, 2021	2	1	0	2	0	2	0	0	​	​	​	​	**7**
Vuurberg, 2018	2	2	0	2	1	2	0	0	​	​	​	​	**9**
Zhu, 2016	2	2	0	2	1	2	0	0	​	​	​	​	**9**
Mean	​	​	​	​	​	​	​	​	​	​	​	​	**7,41 (exc Reilingh**
